# Influence of CNT Length on Dispersion, Localization, and Electrical Percolation in a Styrene-Butadiene-Based Star Block Copolymer

**DOI:** 10.3390/polym14132715

**Published:** 2022-07-02

**Authors:** Ulrike Staudinger, Andreas Janke, Christine Steinbach, Uta Reuter, Martin Ganß, Oliver Voigt

**Affiliations:** 1 Leibniz-Institut für Polymerforschung Dresden e.V., Hohe Str. 6, 01069 Dresden, Germany; andy@ipfdd.de (A.J.); steinbach@ipfdd.de (C.S.); reuter@ipfdd.de (U.R.); oliver.voigt@mvtat.tu-freiberg.de (O.V.); 2 Material Research and Testing Institute (MFPA), the Bauhaus-Universität Weimar, Coudraystraße 9, 99423 Weimar, Germany; martin.ganss@mfpa.de

**Keywords:** carbon nanotubes, ball milling, CNT length, block copolymer, CNT localization, CNT dispersion, electrical conductivity, mechanical behavior

## Abstract

This study followed the approach of dispersing and localizing carbon nanotubes (CNTs) in nanostructured domains of block copolymers (BCPs) by shortening the CNTs via ball milling. The aim was to selectively tune the electrical and mechanical properties of the resulting nanocomposites, e.g., for use as sensor materials. Multiwalled carbon nanotubes (MWCNTs) were ground into different size fractions. The MWCNT length distribution was evaluated via transmission electron microscopy and dynamic light scattering. The nanostructure of the BCPs and the glass transition temperatures of the PB-rich and PS phases were not strongly affected by the addition of CNTs up to 2 wt%. However, AFM and TEM investigations indicated a partial localization of the shortened CNTs in the soft PB-rich phase or at the interface of the PB-rich and PS phase, respectively. The stress-strain behavior of the solution-mixed composites differed little from the mechanical property profile of the neat BCP and was largely independent of CNT amount and CNT size fraction. Significant changes could only be observed for Young’s modulus and strain at break and may be attributed to CNT localization and small changes in morphology. For nanocomposites with unmilled CNTs, the electrical percolation threshold was less than 0.1 wt%. As the CNTs were shortened, the resistivity increased and the percolation threshold shifted to higher CNT contents. Composites with CNTs ground for 7.5 h and 13.5 h showed no bulk conductivity but significantly decreased surface resistivity on the bottom side of the films, which could be attributed to a sedimentation process of the grind and thereby highly compressed CNT agglomerates during evaporation.

## 1. Introduction

Nanocomposites with matrices of block copolymers (BCPs) are very interesting in terms of their nanostructured morphologies and the resulting possibilities for the incorporation and selective localization of nanofillers. The use of carbon nanotubes (CNTs) as fillers is based on their exceptional property profile which enables the development of functional materials with special thermal, mechanical, and electrical properties. CNTs have very high tensile strengths [1], exceeding those of steel by a factor of 20, excellent electrical conductivities comparable to copper [2], and very high thermal conductivities [3]. At the same time, they are characterized by low density. Due to their large aspect ratio (length/diameter ratio; up to 10,000), electrically percolating CNT networks can be generated in different polymer matrices even at very low CNT contents [4,5]. By selective localization of CNTs into suitable phase morphologies of immiscible blends, electrical, thermal, and mechanical properties can be specifically adjusted [6]. As immiscible blends with co-continuous structure have been found to be promising candidates as sensor materials for gas sensing applications by using the double percolation concept [7,8,9], microphase separated block copolymer nanostructures similarly can act as a template for selective localization of nanofillers and thus to form a conductive, sensitive network through the polymer. In the past, there have been several fundamental investigations to selectively introduce CNTs into a phase of BCP [10,11,12,13,14,15,16]. In order to increase the compatibility between the BCP phase and nanofiller and to enable selective placement of CNTs, in the mentioned studies, CNTs were modified with appropriate functional groups. In this context, the localization succeeded only in lamellar and cylindrical structures.

In our own studies on the dispersibility and localization of CNTs in a styrene–butadiene-based star block copolymer [17], non-functionalized multiwalled carbon nanotubes (MWCNTs) as well as MWCNTs functionalized with monoamino-terminated polystyrene and n-octadecylamine were dispersed by solution mixing, but no preferential localization could be demonstrated. In corresponding physical polystyrene/polybutadiene (PS/PB) blends, the non-functionalized and functionalized CNTs showed a clear affinity for the PS phase or the CNTs were localized at the interface of both phases, respectively [17], although viscosity differences of the two homopolymers used may also influence the localization behavior and possibly prevent incorporation in the PB phase. Localization of CNTs in a nanometer-small BCP domain are also hindered by the large aspect ratio (length to diameter ratio) of CNTs, which with their average length of 1.5 µm significantly exceed the domain sizes of the block copolymer phases and can only be embedded when aligned parallel to the domain boundary. In linear styrene-b-styrene-co-butadiene-b-styrene triblocks, ball-milled MWCNTs (length (D50) ≈ 500 nm) were dispersed by melt blending, and TEM images suggested affinity or partial localization of CNTs in the PB-rich phase [18].

To facilitate selective mixing of CNTs into block copolymers, it is useful to use short CNTs, although it should be noted that as the aspect ratio decreases, the electrical percolation threshold increases, i.e., more filler content is required to produce electrically conductive composites.

To shorten CNTs, various strategies have been reported and were summarized in the review of Dai and Wang [19], for example: physical methods such as ball milling, ultramicrotome, or high impact; chemical cutting by the use of oxidizing chemicals which break the carbon bonds; and electrical cutting methods such as electron beams or Jule heat. Raston and coworkers found another interesting method to shorten CNTs in a vortex fluid device under continuous flow conditions using laser irradiation, where the lengths can be controlled via laser pulse energy [20,21,22].

This study focused on shortening CNTs by ball milling as a simple physical cutting method where the CNT length can be controlled by adjusting the milling ball diameter, the amount of powder used, the frequency, and the milling time.

Pierard et al. [23] described the competing effect between the shortening and agglomeration of single-walled CNTs (SWCNTs) during the milling process, which leads to a stabilizing minimum diameter of the aggregates after a certain milling time. Liu et al. [24] found that MWCNTs exhibited increased hydrogen adsorption due to shortening by ball milling, which was attributed to the increase in defects, open nanotube ends, and increased surface area after milling.

Krause et al. [25] investigated the influence of CNT shortening of commercially ball-milled MWCNTs for 5 or 10 h on their length distribution and their dispersibility in aqueous media and on the electrical percolation in melt-mixed polycarbonate/CNT composites. The ball-milled CNTs were shortened to 54% and 35% of their initial length, respectively, and oxygen groups were detected on their surface. The dispersibility was deteriorated in both aqueous media and polymer melt, and the percolation threshold in the composites was slightly increased.

In this work, first, the effect of milling time on CNT length distribution was investigated. Second, the shortened CNTs were incorporated in different proportions into a styrene–butadiene-based star block copolymer using a solution mixing process. The dispersion of the CNTs in the block copolymer, their interactions with the nanodomains, the electrical percolation, and the mechanical behavior were studied.

## 2. Materials and Methods

### 2.1. Materials

Commercially available multiwalled carbon nanotubes (MWCNTs) NC7000^TM^ with a mean diameter of 9.5 nm and a mean length of 1.5 µm were purchased from Nanocyl (Sambreville, Belgium). The MWCNTs exhibit a carbon purity of 90% and a surface area of 250–300 m^2^/g [26]. As a matrix polymer, a styrene–butadiene (SB)-based star block copolymer (Styrolux, supplied by INEOS Styrolution Group GmbH (Frankfurt am Main, Germany) was used. The synthesis of the block copolymer (BCP) is described elsewhere [27]. The overall polystyrene (PS) content is about 75 wt%. The BCP has an asymmetric star architecture with a random poly(styrene-co-butadiene) core containing about 15 wt% of PS, and three short and one long PS arm having an approximate molecular weight M_n_ (GPC, PS standard) of 86 kg/mol and a polydispersity of PD ≈ 2.1 [28]. For the polymer/CNT solution mixing process, the solvent toluene, and for analytical purposes, chloroform were used, both purchased from Merck KGaA (Darmstadt, Germany).

### 2.2. Shortening and Characterization of CNTs

#### 2.2.1. Ball Milling

The MWCNTs were shortened into different size fractions using a mixer mill MM40 from Retsch GmbH, Haan, Germany. For the grinding procedure, 0.5 g of the starting material NC7000 was placed in a 50 mL stainless steel grinding jar. An amount of 10 g of steel balls with a diameter of 5 mm was added. At a frequency of 15 Hz, the grinding time varied from 1.5 h, 3 h, 4.5 h, 6 h, 7.5 h, to 13.5 h. The shortened CNTs are denoted as BM_xh, where BM stands for ball-milled and x represents the grinding time in hours (h).

#### 2.2.2. Surface Characterization of CNT Powder

Scanning electron microscopy (SEM) was applied to characterize the ground CNT powders using a ZEISS Ultra Plus microscope (Carl Zeiss GmbH, Jena, Germany) with a field emission cathode combined with an SE2 detector.

#### 2.2.3. Evaluation of CNT Length Distribution

To evaluate the length distribution of the ground CNTs, a method developed by Krause et al. [29] was used. A total of 1 mg of CNTs was dispersed in 10 mL of chloroform in an ultrasonic bath Bandelin Sonorex (Bandelin electronic GmbH & Co. KG, Berlin, Germany) for 7 min. The dispersion was dropped onto a TEM grid and dried under ambient conditions. A LIBRA^®^ 120 transmission electron microscope (TEM) with a voltage of 120 kV (Carl Zeiss AG, Oberkochen, Germany) was used to take images of the CNTs with 8000× magnification. The length of CNTs was measured in TEM images. The accuracy of magnification calibration was ≈3% and the elliptic distortion of the images was ≈6%. In each case, several image sections were chosen to overlap each other slightly in order to capture the full length of the CNTs. Using the Stream Motion software, the images were combined to be congruent accordingly. The CNT lengths were measured with the help of the polyline function. The number of measured CNT lengths was dependent on the dispersion quality and availability of the CNTs on the TEM grid and varied between 150 and 290. To characterize the length distribution, the 90%, 50%, and 10% quantiles were calculated, indicating that 90, 50, or 10% of all measured lengths were less than the quantile, respectively. The quantiles were named D90, D50, and D10, respectively.

#### 2.2.4. Dynamic Light Scattering

CNTs BM_7.5h and BM_13.5h were ground to such an extent that no CNTs adhered to the TEM grid and thus no length evaluation could be carried out. Therefore, for both size fractions and comparatively for CNTs BM_6h, dynamic light scattering was applied for particle size determination. The CNTs were dispersed in chloroform and treated for 5–10 min in an ultrasonic bath. The experiment was performed according to ISO 22412 using a Zetasizer Nano ZS. At least two determinations per CNT size fraction were performed. The CNT dispersion was exposed to monochromatic laser light. The Brownian molecular motion of the particles in the solution causes minimal change in the wavelength of the scattered light and thus small variations in intensity. The magnitude of these fluctuations provides information about the diffusion velocity of the particles and thus about their size.

#### 2.2.5. Sedimentation Analysis of CNTs in Polymer Solutions

To characterize the sedimentation behavior of the CNTs in polymer solutions which can significantly influence the quality of CNT dispersion and network formation in the solvent cast films, a centrifugal separation analyzer (CSA) LUMiSizer 6100-29 (L.U.M. GmbH, Berlin, Germany) was used. Polymer/CNT dispersions containing 1 wt% of CNTs referred to the added polymer were prepared in the same way as described in Section 2.3.1. The sedimentation behavior was studied by placing a sample of each of the dispersions in a glass cuvette with a cross-section of 10 × 10 mm and by centrifugation for 10 h at a centrifugal speed of 3000 rpm and a temperature of 20 °C. The analytical centrifuge instantaneously measured the absorbance of transmitted light over the entire sample length and evaluated space- and time-resolved extinction profiles of the samples between the bottom and the fluid level of the dispersion. To characterize the dispersion stability, the transmission profiles were integrated between the positions 107 mm and 124 mm, i.e., in the middle segment of the glass cuvette. From the initial slope of the integral transmission, the sedimentation rate of the CNT particles could be determined. A low sedimentation rate corresponds to high dispersion stability and is due to good particle dispersion. At a high sedimentation rate, the particle dispersion is insufficient and remaining larger agglomerates sediment fast. The shape of the transmission profiles characterizes the type of sedimentation. In polydisperse dispersions with very low particle concentration, the profiles consist of horizontal curves. In this case, sedimentation can take place unhindered and the particles sediment due to their size. If the transmission profiles consist of parallel vertical curves, the dispersions are either monomodal dispersions, in which rigid spherical particles of the same size and density migrate in the centrifugal field [30], or dispersions with higher particle concentrations, in which agglomerates and networks form that hinder each other and sediment faster than the primary particles.

### 2.3. Processing of BCP/CNT Composites

#### 2.3.1. Solution Mixing

BCP/MWCNT composites of each CNT size fraction and of neat NC7000 were prepared by solution mixing using an experimental procedure that has been described in an earlier publication [17]. A total of 1 wt% of the polymer was dissolved in 20 mL of toluene for 30 min with the help of a magnetic stirrer. Then, 0.1 to 2.0 wt% of MWCNTs (concentration related to the polymer) were pre-dispersed in 20 mL of toluene for 10 min by ultrasonic treatment using an UP400S processor (Hielscher Ultrasonics GmbH, Teltow, Germany) having a maximum frequency of 24 kHz and a power of 400 W. A standard sonotrode (H3) with a tip diameter of 3 mm was used and the amplitude was set to 50%. Subsequently, the polymer solution was added to the CNT dispersion, mixed for 30 min via magnetic stirring, and was treated by ultrasound for another minute. The final dispersions were poured into a Petri dish to slowly evaporate the solvent over 3–5 days. The free-standing films exhibited a film thickness of ≈0.3 mm and were additionally dried in a vacuum chamber at 40 °C for 24 h.

#### 2.3.2. Dip Coating

To study the localization of shortened CNTs in the nanostructured BCP morphology via atomic force microscopy, thin films of BCP/MWCNT composites containing 1.5 wt% and 2 wt% of CNTs BM_ 4.5h were additionally prepared on silicon (Si) wafers via dip coating. Si wafers were cleaned according to the RCA standard, applying a pre-cleaning step with absolute ethanol in an ultrasonic bath for 20 min. Cleaning was conducted by stirring the vessel with wafers for 20 min in a solution mixture of hydrogen peroxide, ammonium hydroxide, and Millipore water in the ratio of 1:1:5 at a temperature of 70 °C. After removing the hot cleaning solution, the wafers were repeatedly rinsed with Millipore, two times shortly and three times for 5 min. Finally, the substrates were blown dry with nitrogen. The polymer solution was prepared by dissolving 1 wt% of the BCP in 10 mL of chloroform, applying magnetic stirring for 1 h. Thin BCP films were produced by vertically dipping the wafer into the polymer solution and the immediate pull-out of the wafer at two different pull-out speeds of 1.5 mm/s and 2.5 mm/s in order to vary the layer thickness. To prepare the BCP/MWCNT composite films, 1.5 wt% CNTs were first added to the polymer stock solution and dispersed by an ultrasonic processor for 5 min. Subsequently, the composite films were dip coated in the same way as described above for the polymer films. Then, another 0.5 wt% of MWCNTs was added to the dispersion so that the total content was about 2 wt%. Dispersion of the CNTs and film preparation were again carried out analogously. The film thickness of the dip coated samples was analyzed using spectroscopic ellipsometry (alpha-SE, J. A. Woollam Co. Inc., Lincoln, NE, USA). To fit the ellipsometric data, a box model of silicon substrate, silicon dioxide, polymer, and a roughness layer was used [31]. The optical dispersion of the polymer layer was modeled by a Cauchy function (n(λ) = A + B/λ^2^, k = 0), and the roughness layer by an effective medium approximation (Bruggeman EMA) [32] with 50% polymer and 50% void. The dispersions of silicon and silicon dioxide were taken from the database (CompleteEASE, J. A. Woollam Co. Inc., Lincoln, NE, USA). For polymer films with CNT content, thickness non-uniformity was considered in the optical fit.

### 2.4. Evaluation of CNT Macrodispersion

To characterize the dispersibility of the different CNT size fractions in the BCP matrix, transmission light microscopy of CNT/BCP composites with 1 wt% of MWCNTs was conducted with an Olympus-BH2 microscope (Olympus Deutschland GmbH, Hamburg, Germany). For this purpose, thin slices with a thickness of 5 µm were cut from the cross-section of the free-standing solution cast films with a Leica RM 2265 microtome, which is equipped with a liquid nitrogen device LN22 (Leica Microsystems GmbH, Wetzlar, Germany) cooling the sample down to 0 °C. The cuts were applied to glass slides and attached with the aqueous mounting medium Aquatex^®^. To evaluate the agglomerate area ratio of CNTs in the polymer matrix, ten representative images of each composite sample were taken. The area of non-dispersed CNTs was determined using the Stream Motion evaluation software and was divided by the total area of the images to obtain the agglomerate area ratio *A*, expressed in (%). CNT agglomerates with minimum areas of 1 µm^2^, 5 µm^2^, 10 µm^2^, and 20 µm^2^ were considered.

### 2.5. Characterization of Composite Morphology and CNT Localization

To study the BCP phase morphology, CNT dispersion and the interactions between CNTs and BCP phases, transmission electron microscopy (TEM) and atomic force microscopy (AFM) were used. For TEM (LIBRA^®^ 120, Carl Zeiss AG, Oberkochen, Germany), ultrathin cross-sections of ≈60 nm were cut from selected solvent cast films using an EM UC6/EM FC6 ultramicrotome (Leica Microsystems, Wien, Austria) at a temperature of −160 °C. The cuts were transferred to a carbon-coated copper grid and stained with osmium tetroxide. The heavy metal accumulates in the PB-rich phase, which leads to the formation of a contrast between the BCP phases in the TEM.

AFM investigation was conducted on microtomed surfaces of selected solution cast films and on dip coated films in the tapping mode by Dimension FASTSCAN (Bruker-Nano, Billerica, MA, USA). For the measurements of the microtomed surfaces, silicon nitride SPM sensors with integrated silicon tip FASTSCAN-C (Bruker-Nano, Billerica, MA, USA) with a spring constant of nominal 0.8 N/m, a resonance frequency of nominal 300 kHz, and a tip radius of 5 nm were used. For the dip coated films, silicon nitride-SPM-sensors with integrated silicon tip SCANASYST-FLUID+ (Bruker-Nano, Billerica, MA, USA) with a spring constant of nominal 0.7 N/m, a resonance frequency of nominal 150 kHz, and a tip radius of 2 nm were applied. Height images (surface morphology) and phase images were taken simultaneously. According to Magonov [33], the scan conditions were chosen to obtain a stiffness contrast (free amplitude >100 nm, set-point amplitude ratio 0.8). This means that brighter areas in the phase image are stiffer areas.

### 2.6. Dynamic Mechanical Analysis

The phase behavior and the dynamic glass transition of the BCP nanocomposites were characterized by dynamic mechanical analysis (DMA) in the temperature range of −65 °C to 130 °C. Measurements were performed on test specimens with dimensions of 30 × 8 × 0.3 mm^3^ in tension mode using a DMA GABO Eplexor 500 dynamical mechanical analyzer equipped with a 25 N load cell and an oven (Netzsch Gerätebau GmbH, Selb, Germany). The tests were carried out strain-controlled with a static strain of ε_stat_ = 0.1% and a dynamic strain of ε_dyn_ = 0.08% by applying a sinusoidal load at a frequency of 10 Hz. The sinusoidal displacement and force signal were checked during the measurement. The heating rate of the temperature ramp tests was 1 K/min. All specimens were clamped with the same torque and were cooled down to the initial temperature of −65 °C with a defined preload of 0.1 N. Prior to testing, the samples were held at a temperature of −65 °C for 30 min to reach the thermal equilibrium. At least two specimens were tested for each composition. The storage modulus (*E*′), loss modulus (*E*″), and tan delta (*tan δ*) were evaluated by considering 40 stress-strain cycles at a defined temperature using the software Eplexor 9 (Netzsch Gerätebau GmbH, Selb, Germany).

### 2.7. Mechanical Properties

Tensile tests were performed on solution cast films having a thickness of ≈0.3 mm using a universal testing machine UPM Zwick 1456 (ZwickRoell GmbH & Co. KG, Ulm, Germany) equipped with a MultiXtens extensometer using testing standard DIN EN ISO 527-2/S3a/50. The test speed was set at 50 mm/min, and Young’s modulus was determined at a speed of 1 mm/min. At least five dog-bone-shaped specimens of each sample with a geometry of 50 × 4 × 0.3 mm^3^ were tested to study the stress-strain behavior of the BCP/CNT composites in dependence on the CNT content and on the CNT length.

### 2.8. Electrical Characterization

#### 2.8.1. Powder Conductivity of CNTs

A homemade powder conductivity measuring cell, developed by IPF Forschungs-technik, was used to evaluate the electrical conductivity and density of the CNT powders at different pressures. The device is combined with an analysis software based on Agilent VEE Version 9.3 (Santa Clara, CA, USA) to record the measurement data. A detailed description of the method can be found elsewhere [34]. The powder density and conductivity were determined at increasing pressures from 5 MPa to 30 MPa. The measurement was carried out to consider the possible influence of filler conductivity on the electrical conductivity of the composites.

#### 2.8.2. Volume Resistivity of BCP/CNT Composites

The electrical volume resistivity (Ω∙cm) of solution cast films was determined with the use of a Keithley electrometer 6517A (Keithley, Cleveland, OH, USA). A connected Keithley test fixture 8009 was used for samples with resistivity >10^7^ Ω∙cm. The voltage was set to 40 V. For conductive samples with a volume resistivity <10^7^ Ω∙cm, a self-made test fixture (developed by IPF Forschungstechnik, Leibniz-Institut für Polymerforschung Dresden e.V., Dresden, Germany) with two in-plane gold electrodes and a voltage of 4 V was applied. Ten individual measured values were automatically recorded and averaged for each measurement.

#### 2.8.3. Surface Resistivity of BCP/CNT Composites

For electrical surface resistivity measurements, a Hiresta and a Loresta electrometer (Mitsubishi Chemical Analytech Co., Ltd., Yamato, Japan) were used. Hiresta was connected to a ring electrode (URS) with an outer diameter of 18 mm, and it has a measurement range of 10^4^ Ω to 10^13^ Ω. It was applied for samples with a resistance >10^7^ Ω. Depending on the resistance of the sample, the applied voltage was varied from 10 V to 500 V. Loresta was equipped with a four-point electrode (ESP) with pin distances of 5 mm and pin diameters of 2 mm. The voltage was set to 10 V. It has a measurement range of 10^−3^ Ω to 10^10^ Ω and was used for samples with a resistance <10^7^ Ω. Five measurements were conducted on the top and bottom side of each sample to evaluate the arithmetical average and the standard deviation. The unit of surface resistivity is specified in Ohm per square (Ω/sq.).

## 3. Results and Discussion

### 3.1. Characteristics of Shortened CNTs

**Figure 1:**
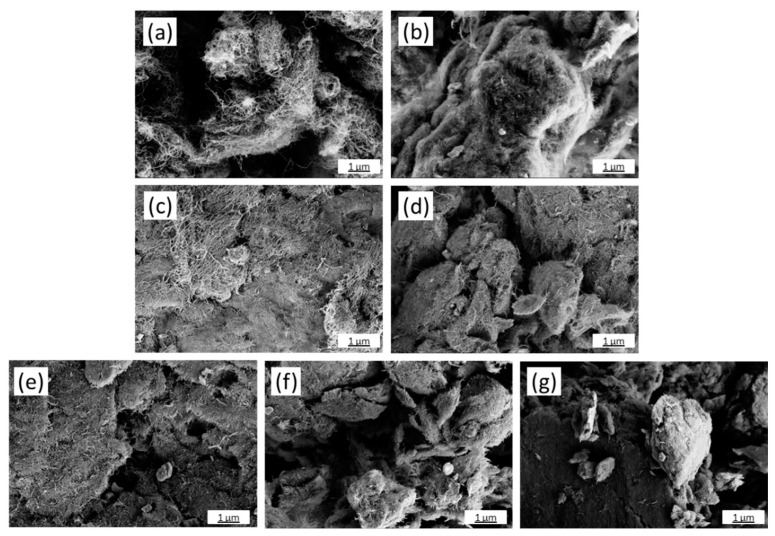
SEM images of CNT powders: neat NC7000 (**a**) and CNTs ground for 1.5 h (**b**), 3 h (**c**), 4.5 h (**d**), 6 h (**e**), 7.5 h (**f**), and 13.5 h (**g**).

The characteristics of the shortened CNTs were analyzed using SEM. Micrographs of neat NC7000 and ground CNTs at high magnification are presented in Figure 1. SEM images at lower magnifications are documented in the Supplementary Materials (Figure S1). The original NC7000 powder exhibited fluffy agglomerates which were loosely interconnected.

In Figure 1a, long CNTs can be observed which are networked in a loose tangle structure. After ball milling for 1.5 h, the CNT agglomerates appeared more compact and flatter, more like platelets (Figure S1b), which was due to the impact forces of the steel balls during milling. The CNT network appeared strongly matted (Figure 1b). With increased grinding time, the agglomerates became more and more compacted. Up to a grinding time of 4.5 h, larger CNT network structures were still visible (Figure 1c,d). At higher grinding times of 6 h to 13.5 h, the visible CNTs appeared significantly shortened, since only small CNT tips protruded from the very compacted agglomerate surfaces (Figure 1e–g).

**Figure 2:**
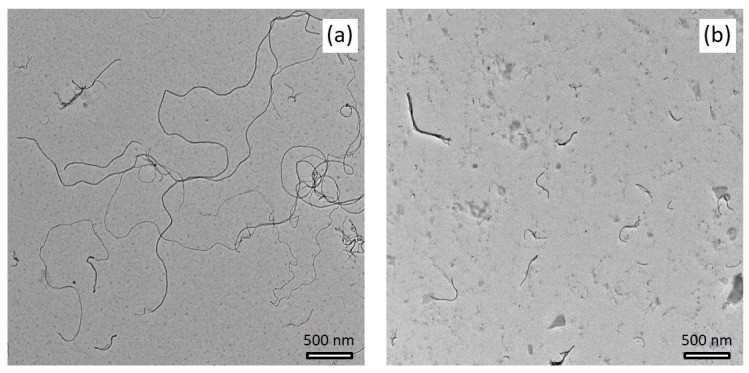
TEM images of shortened CNTs: (**a**) BM_1.5h; (**b**) BM_6h.

**Figure 3:**
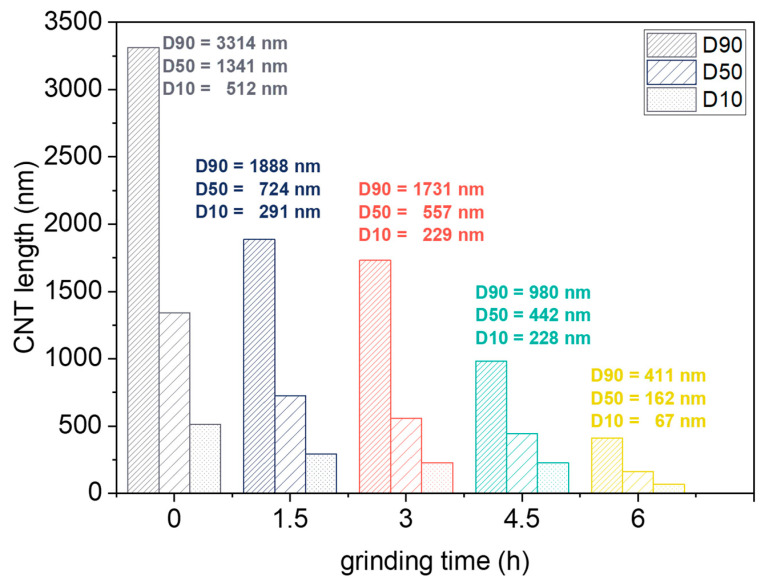
CNT length distribution as a function of grinding time [25].

**Figure 4:**
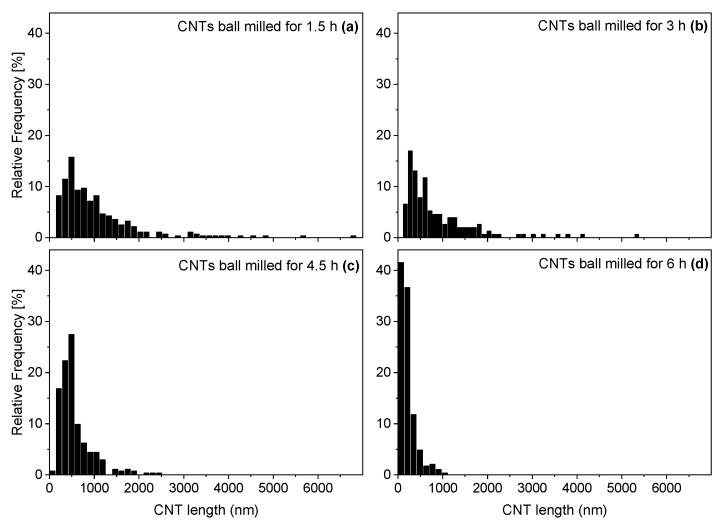
Histograms of CNTs ground for 1.5 h (**a**), 3 h (**b**), 4.5 h (**c**), and 6 h (**d**).

The length evaluation of CNTs ground for 1.5 h up to 6 h revealed a significant shortening effect as shown in exemplary TEM images in Figure 2, comparing CNTs BM_1.5h and BM_6h. Quantitative values from TEM image analysis of CNT length (i.e., D10, D50, and D90) versus CNT ball milling time are illustrated in the diagram of Figure 3, including neat NC7000. The histograms in Figure 4 present the length distribution of the ground CNTs.

While the D50 value for the CNT initial length was 1341 nm [25], a significant shortening to a D50 value of 762 nm already occurred after 1.5 h (Figure 3). After 6 h, the D50 value was only 162 nm. Up to a grinding time of 3 h, the length distribution remained relatively broad and then became significantly narrower up to a grinding time of 6 h, as shown by the histograms in Figure 4. CNTs ground for 7.5 h and 13.5 h could not be deposited on the TEM grid, probably because the isolated CNTs were too small or compact agglomerates of the heavily ball-milled CNTs did not adhere to the grid. Therefore, particle size determination by dynamic light scattering was performed on these CNT powders, and as a reference, also on CNTs ground for 6 h.

The polydispersion index (PDI) is a measure of the particle size distribution and a large PDI (>0.5) indicates a broad particle size distribution, as in the case of the measured CNT fractions (Table S1, Supplementary Materials). In all samples, sedimentation of the particles was observed during the measurement, which leads to a reduced significance of the fits. The fits of the cumulative analysis and the distribution analysis become inaccurate in the range of large particles. The cumulative analysis is more suitable for monodisperse distributions and is no longer used for particles with PDI > 0.7. Therefore, only the results of the distribution analysis were considered here, whereby the values of the large particles are to be regarded critically. Measurement results of the particle size distribution analysis of DLS evaluation for all measured samples are given in Table S1 (supplementary materials).

**Figure 5:**
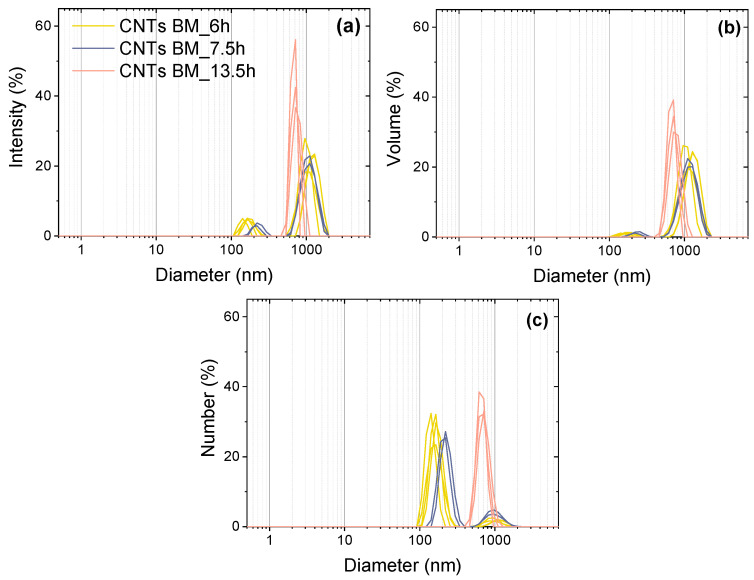
Particle size distribution of ground CNTs weighted by intensity (**a**), volume (**b**), and number (**c**) determined by DLS (chart legend is valid for all diagrams).

From the intensity-weighted particle size distribution (Figure 5a), the volume (Figure 5b) and number (Figure 5c) distribution were calculated considering a refractive index (RI) of the CNTs of 2.5 and an absorption of the CNTs of 1.0, as well as the standard values (viscosity and RI) for chloroform at 20 °C. Based on the distribution analysis as represented by the diagrams in Figure 5, two size distribution curves were identified for each of the 6 h and 7.5 h milled CNTs. Once, very small particles occurred, with average sizes of 140–170 nm for 6 h milled CNTs and 210–240 nm for 7.5 h milled CNTs. Large particles had an average size of 940–1300 nm for 6 h milled CNTs and 1000–1200 nm for 7.5 h milled CNTs. The number of smaller particles clearly predominated and reflects the presence of isolated CNTs. Larger particle sizes indicate the existence of CNT agglomerates. In the case of CNTs ground for 13.5 h, only one distribution curve was observed, with an average particle size of 660–770 nm. The distribution curve was relatively narrow.

**Figure 6:**
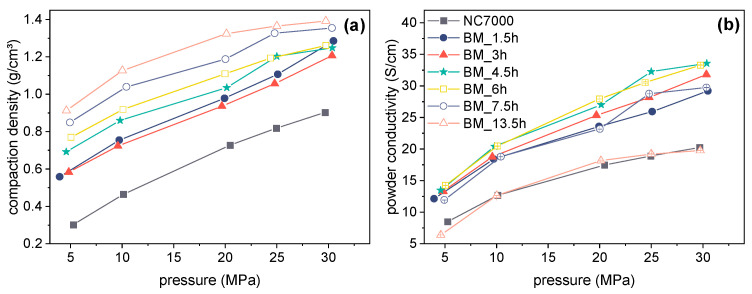
Compaction density (**a**) and powder conductivity (**b**) of CNTs as a function of pressure (chart legend is valid for both diagrams).

For further characterization of the influence of ball milling on the characteristic of the CNT powder, the compaction density and the electrical conductivity were studied using a special conductivity measuring cell under defined pressure. Figure 6 shows the compaction density and powder conductivity of the CNTs as a function of pressure for the NC7000 and different grinding times. As expected, with increasing pressure, the compaction density rose for the different CNT size fractions (Figure 6a). However, the compaction density also increased with increasing grinding time of the CNTs, which indicated an enhanced compression of the CNT agglomerates during grinding and resulted from the shortening of the CNTs, since powders with smaller particles can be compressed better than powders with large particles. This was also reflected in an increase in pouring density of the milled CNT powders that was observed. With increasing pressure, the electrical powder conductivity increased for all CNT powders (Figure 6b) due to enhanced contacts between the particles. With increasing grinding time, the powder conductivity initially measured at a pressure of 30 MPa increased significantly compared to the starting powder NC7000 (20.2 S/cm), with the highest conductivities for CNT powders occurring after a grinding time of 4.5 h (33.6 S/cm) and 6 h (33.3 S/cm). For CNTs BM_13.5h, the powder conductivity dropped significantly (19.2 S/cm) and was around the value for untreated NC7000.

### 3.2. Stability of Polymer/CNT Dispersions

BCP/CNT dispersions were characterized by applying centrifugation forces to study the stability of the dispersions and the sedimentation behavior of the CNTs in the polymer–toluene solutions. The transmission profiles of the BCP/CNT dispersions are shown in Figure S2, Supplementary Materials. The profiles exhibited vertical curves at the beginning of the centrifugation and horizontal curves in the later course when NC7000 and up to 3 h ground CNTs were included. For dispersions containing CNTs ball-milled for 4.5 h or longer, the profiles consisted primarily of horizontal profiles. The horizontal profiles indicate a polydisperse distribution of CNT particle sizes. The vertical curves illustrate the initial strong sedimentation of large CNT agglomerates of similar sizes.

**Figure 7:**
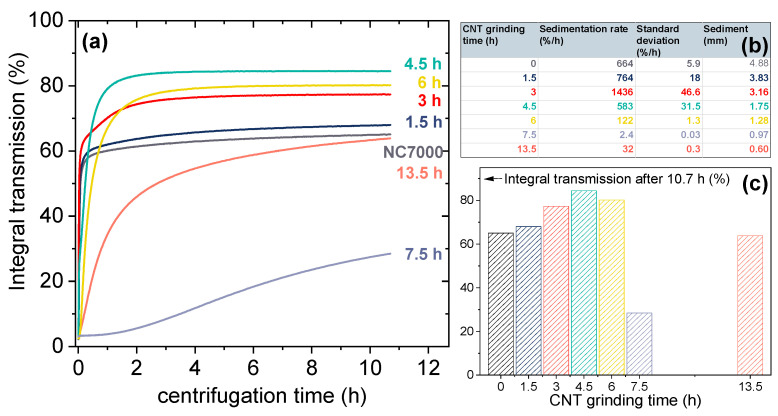
(**a**) Integral transmission of BCP/CNT dispersions as a function of centrifugation time; (**b**) sedimentation rate and sediment height of CNTs in BCP–toluene solutions; (**c**) integral transmission of BCP/CNT dispersions after 10.7 h of centrifugation.

The integral transmission versus centrifugation time, which was calculated by the integration of the transmission profiles between positions 107 mm and 124 mm, for different ball milling times and NC7000 is shown in Figure 7a. The sedimentation rate (%/h) of the CNT particles could be calculated from the initial slope of the integral transmission curve and initially increased significantly with increasing grinding time from 664 %/h (NC7000) to 1436 %/h (BM_3h) (Figure 7b). Further increase in CNT grinding time led to a distinct decrease in the sedimentation rate, exhibiting a minimum of 2.4 %/h for dispersions containing CNTs BM_7.5h. A low sedimentation rate indicates significantly enhanced dispersion stability, which was the case for BCP/CNT dispersions containing CNTs ground for 4.5 h to 13.5 h.

The formation of a sediment during centrifugation is demonstrated by the photographs in Figure S3, Supplementary Materials, showing the dispersions after centrifugation. The height of the sediment (in mm) was measured from the transmission profile diagrams between the liquid–solid phase boundary (drop of the last profile curve) after the position of 124 mm and the transition to the cuvette bottom (peak of the final rising curve) and is displayed in the table in Figure 7b. The height of the sediment continuously decreased with progressive grinding time of the CNTs and was mainly dominated by the equally decreasing bulk density of the CNT powders as a function of the grinding time (see Figure 6).

The integral transmission of BCP/CNT dispersions after 10.7 h of centrifugation increased with increasing CNT grinding time up to 6 h compared to the pure NC7000 (Figure 7b). On the one hand, the CNTs were shortened by the grinding process, but on the other hand, the originally very loose fluffy CNT network of the NC7000 was considerably compressed as indicated by the SEM pictures of the CNT powders (Figure 1). This resulted in many compact agglomerates that sediment more quickly. Additionally, as the grinding time of the CNTs increased, the sedimentation process was completed later than for pure NC7000. The integral transmission curve reached its plateau only with longer centrifugation times. If more particles sediment, this results in a higher final integral transmission.

However, if the CNTs were ground for 7.5 h, the sedimentation behavior was fundamentally different. The CNTs were then predominantly very stable in the polymer solution, which was reflected in the low integral transmission and the dark grey dispersion as shown in Figure S3, Supplementary Materials. Strong compression during further grinding of the CNTs up to 13.5 h led to the formation of larger, very compact agglomerates again and resulted in an increase in the sedimentation rate and in the integral transmission (Figure 7c).

### 3.3. BCP/CNT Nanocomposites: CNT Dispersion and BCP Morphology

#### 3.3.1. CNT Macro- and Microdispersion

**Figure 8:**
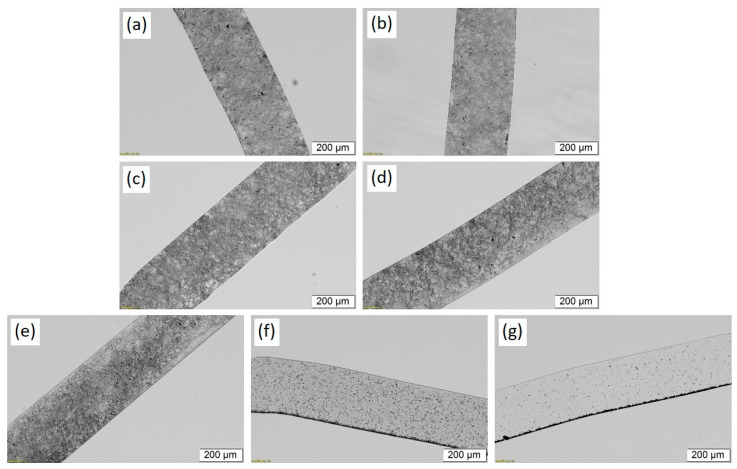
Transmission light microscopy images of 5 µm thin slices of BCP/CNT solution cast films containing 1 wt% of (**a**) neat NC7000, (**b**) BM_1.5h, (**c**) BM_3h, (**d**) BM_4.5h, (**e**) BM_6h, (**f**) BM_7.5h, and (**g**) BM_13.5h.

**Figure 9:**
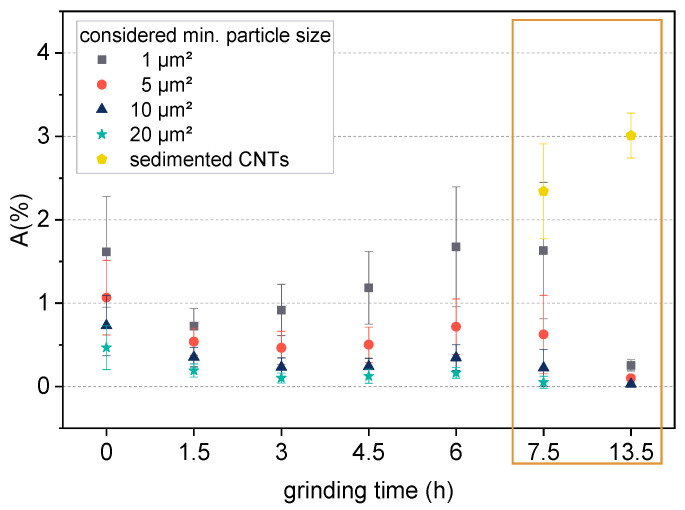
Agglomerate area ratio *A* in solution cast BCP/CNT composites with 1 wt% of CNTs as a function of grinding time. The considered minimum agglomerate particle size varied between 1 µm^2^ and 20 µm^2^. For composites containing CNTs BM_7.5h and BM_13.5h, *A* of the sedimented particles was additionally calculated.

The transmission light microscopy (TLM) images (Figure 8) and the agglomerate area ratio analysis from the microscopy images in (Figure 9) provide information on the distribution and dispersion of CNT agglomerates in solution cast films of BCP/CNT composites. Composites with 1 wt% CNTs were selected for analysis. TLM images illustrated that for composites with 1 wt% NC7000 and with CNTs ground up to 6 h the CNTs were well-dispersed in the polymer matrix (Figure 8a–e). The agglomerate area ratio *A* was already very low for films with neat NC7000 (Figure 9). If only large particles of at least 20 µm^2^ in size were considered, *A*_20µm^2^_ = 0.5, and if particles of 1 µm^2^ or larger were considered, *A*_1µm^2^_ = 1.6 (Figure 9). With increasing grinding time, the fraction of larger agglomerates decreased slightly. For composites with 1 wt% BM_4.5h, the agglomerate area ratio was *A*_20µm^2^_ = 0.1. If particles from 1 µm^2^ were considered, a minimum of *A*_1µm^2^_ = 0.7 occurred for composites containing BM_1.5h. With increasing grinding time, the agglomerate area ratio in the composites increased again and was back at the value for NC7000 for BCP/CNT with BM_6h. This was due to the increasing compression of the CNT particles during grinding, which competed with the shortening of the CNTs. These compressed particles are difficult to separate during ultrasonic treatment.

In composite films with CNTs ground for 7.5 h and 13.5 h, an accumulation of CNTs occurred at the bottom of the film, while the examined film sections appeared significantly more transparent than those of the composites with CNTs ground for shorter times (Figure 8f–g). As the solvent slowly evaporates during film formation, sedimentation of CNT agglomerates takes place, which was more pronounced for BM_13.5h than for BM_7.5h (Figure 9). These observations seem to be in contrast to the results of the stability studies of the CNT dispersions in Section 3.2, where a low sedimentation rate and a low integral transmission were found for BCP/CNT dispersions with BM_7.5h and BM_13.5h, indicating a high stability of the dispersions. The photographs of the centrifuged dispersions (Figure S3) showed homogeneous gray dispersions, indicating a very good and stable dispersion of CNTs in the polymer solution. However, the dispersions also exhibited a sediment at the bottom of the cuvette after centrifugation (see Figure S3 and Figure 7b). This was obviously a fraction of highly compressed CNT agglomerates with high density which sedimented on the bottom of the polymer solution also during the evaporation process. The velocity of CNT agglomerate sedimentation in the polymer solution during the evaporation process obviously was distinctly increased for CNTs ground for 7.5 h and 13.5 h. The sedimentation velocity of a particle can be determined with the help of Stokes’ law and is governed by the forces acting on the particles such as buoyancy, gravity, friction, or Brownian motion (hydrodynamic forces) by the particle–particle interactions, which depend on the distances between the particles and the particle surface properties and by the interactions with the medium [35]. With increasing particle diameter and increasing density difference between particle and liquid, the sedimentation velocity of the particle increases. Thus, the sedimentation of the compact agglomerates of BM_7.5h and BM_13.5h can be related to their high density and their poor interaction with the polymer and the solvent. The CNT sedimentation affected the electrical properties of the films, which is discussed in Section 3.5.

**Figure 10:**
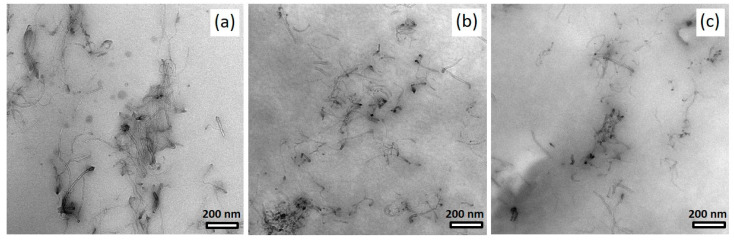
TEM images of specimens of BCP/CNT composites containing 1 wt% of neat NC7000 (**a**), CNT BM_3h (**b**), and CNT BM_6h (**c**); samples were not stained with OsO_4_.

TEM images in Figure 10 provide information on CNT dispersion in the micro- to nanometer range of selected BCP/CNT composite samples with 1 wt% NC7000 and 3 h and 6 h ground CNTs, respectively. The block copolymer morphology of these samples, which were not stained with OsO_4_, is not visible in the TEM images. In films containing the neat NC7000, the CNTs were partly dispersed and partly in bundles as well as in loose agglomerates. The CNTs were well-interconnected due to their lengths (Figure 10a). In composites containing MWCNTs ground for 3 h and 6 h, the CNTs were, as expected, significantly shorter and more separated and agglomerates tended to be smaller (Figure 10b–c).

#### 3.3.2. Influence of Shortened CNTs on BCP Morphology and Phase Behavior

The complex morphology of the highly asymmetric SB star block copolymer has been discussed comprehensively in the past [17,27,36,37,38]. Due to the molecular architecture of the BCP consisting of a soft SB core with a soft phase content of ≈40 wt% (and a total PB content of 25 wt%) and one long and three short PS arms, a weakly ordered lamellar morphology exhibiting very short and curved lamellae structures is formed [17]. As very well-illustrated by Thomann et al. [38], the short PS arms are partly completely enclosed by the soft PB-rich phase. In addition, there are lamellar domains formed by the PB-rich phase and the PS phase, which consist of long and short PS arms. The domain size of the PB-rich lamellae is significantly smaller than that of the PS lamellae [38].

**Figure 11:**
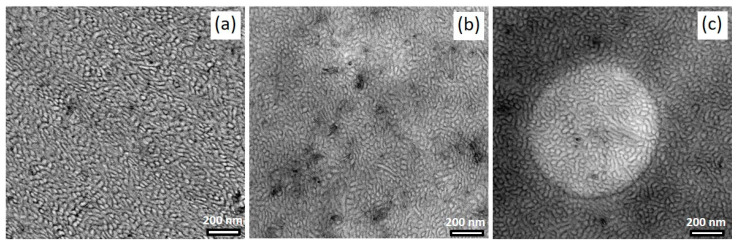
TEM images of BCP morphology containing 1 wt% of neat NC7000 (**a**), CNT BM_3h (**b**), and CNT BM_6h (**c**).

**Figure 12:**
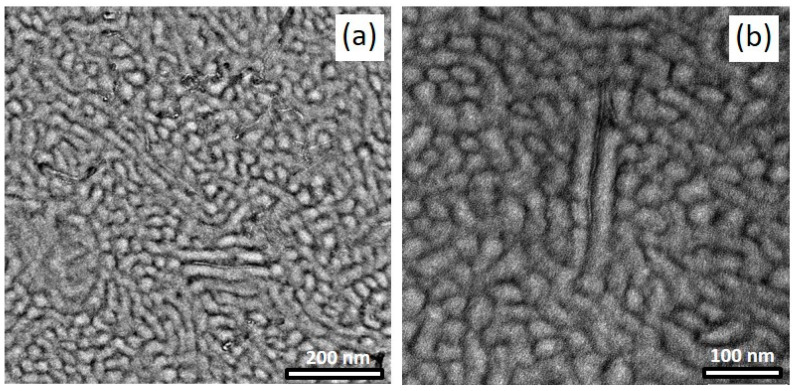
TEM images of CNTs BM_3h (**a**) and BM_6h (**b**) localized in the soft PB-rich domain of the BCP.

TEM images of OsO_4_-stained films are presented in Figure 11, showing the morphology of solution-mixed BCP/CNT composites with 1 wt% NC7000 (a) and 3 h (b) and 6 h milled CNTs (c), respectively. As already found in our previous studies [17,18,39], the BCP morphology seemed not to be strongly affected by the incorporation of CNTs. However, the morphologies of the composites with shortened CNTs tended to be more ordered, with the PS phase appearing to be slightly widened and the PS domain size more uniform (Figure 11). However, this is difficult to quantify due to the complex morphology formation. As shown by the magnified TEM images in Figure 12, shortened CNTs were able to localize in the PB-rich phase (dark) or align along the interface of PB-rich and PS lamellae. The MWCNTs used in this study appeared to interact preferentially with the soft PB-rich phase. With sufficient shortening of the CNTs, they were able to localize directly in the PB phase. From AFM images shown in Figure S4, Supplementary Materials, it is apparent that both the areas with larger agglomerates of neat NC7000s and dispersed shortened CNTs were surrounded by the soft PB-rich phase, i.e., there was an enrichment of soft phase near the CNTs. This effect was not detectable from the TEM images.

**Figure 13:**
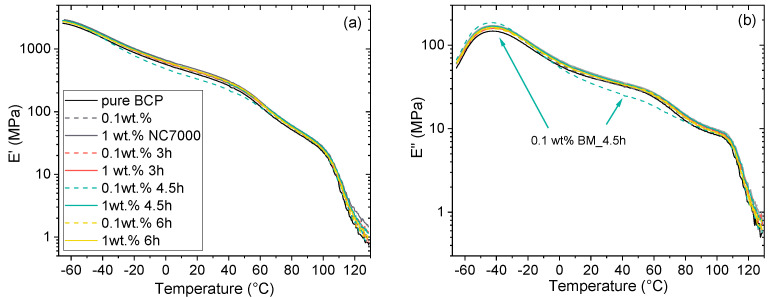
Storage modulus *E*′ (**a**) and loss modulus *E*″ (**b**) versus temperature of BCP/CNT nanocomposites (chart legend is valid for both diagrams).

In our former work, it was observed by dynamical mechanical analysis (DMA) that the incorporation of small amounts of chemically modified nanoplatelets can result in significant changes in the phase behavior of the studied SB-based star block copolymer [40]. In the present study, the influence of the addition of ground CNTs on the phase behavior of the BCP was investigated with DMA using temperature ramp tests. The storage modulus (*E*′) and the loss modulus (*E*″) as function of temperature for the neat BCP and for nanocomposites with 0.1 wt% and 1 wt% NC7000 and CNTs ground for 3 h, 4.5 h, and 6 h are plotted in Figure 13a,b, respectively.

**Figure 14:**
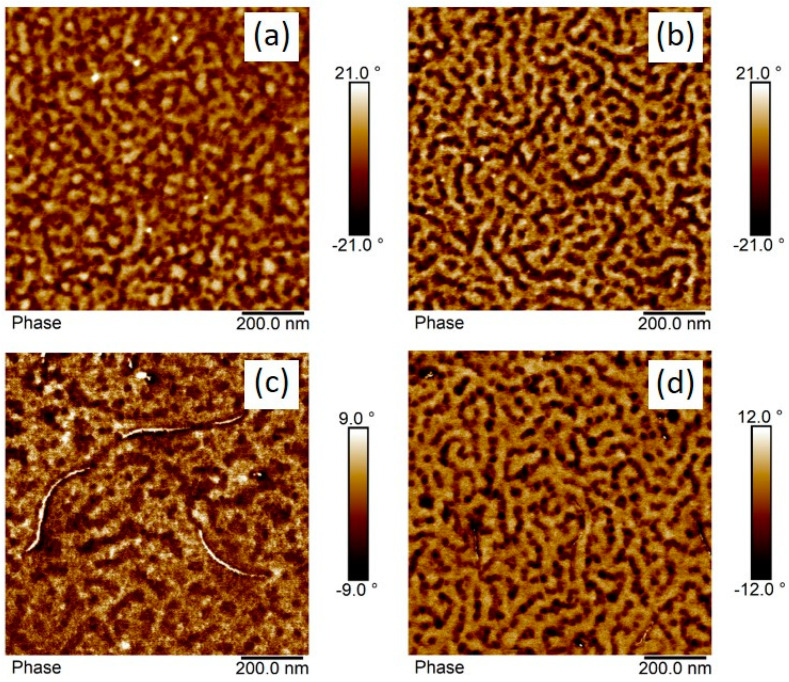
AFM phase images of dip coated films of neat BCP with thicknesses of 37 nm (**a**) and 60 nm (**b**) and BCP with 2 wt% of CNT BM_4.5h with thicknesses of 46 nm (**c**) and 62 nm (**d**).

The star block copolymer matrix showed two characteristic glass transitions corresponding to the Poly(S-co-B) phase (PB-rich phase) and the PS phase as indicated by the significant drop in *E*′ and the local maxima of *E*″ in the range of −55 °C to −30 °C and 90 °C to 110 °C, respectively. The glass transition temperatures of the PB-rich phase and PS phase remained nearly unaffected when incorporating 0.1 and 1 wt% ball-milled CNTs. In addition, Adhikari et al. [41] discussed the possibility of the existence of a third phase for this type of star block copolymer due to miscibility effects between PS chains and Poly(S-co-B) chains in the interphase region. The existence of a third phase or interphase was indicated by a slight drop in *E*′ and the local maxima of *E*″ between 40 °C to 60 °C which could be observed for the neat BCP and all investigated nanocomposites. The incorporation of the CNTs resulted in a slight increase in the storage moduli (*E*′) in the investigated temperature range as shown in Figure 13a with exception of the nanocomposite with 0.1 wt% CNTs BM_4.5h. For the nanocomposite with 0.1 wt-% CNTs BM_4.5h, the storage modulus between −20 to 60 °C was lower than of the pure BCP. The loss modulus as a function of temperature also showed a different behavior for composites with 0.1 wt% CNTs BM_4.5h which exhibited a slightly increased *E*″ peak at the glass transition of the PB-rich phase and a distinct lower *E*″ than all other nanocomposites in the temperature range of −10 °C to 80 °C. These changes are probably associated with increased localization of effectively shortened CNTs in the PB-rich phase and thus increased interactions between the PB-rich phase and the interphase in this nanocomposite, which may cause local changes in morphology. The embedding of the CNTs in the S/B phase or the function of the CNTs as templates for structure formation, i.e., enrichment of the PB-rich phase around the CNTs, is suggested by AFM studies (see Figure S4e–f, Supplementary Materials) and discussed below (Figure 14c).

To further visualize the localization behavior of CNTs in the block copolymer morphology, thin BCP/CNT films with 2 wt% BM_4.5h were dip coated on silicon wafers. The film thickness was affected by varying the pull-out speed (dip coating velocity). This had an influence on the morphology formation of the block copolymer. As discussed by Pospiech et al. [42], film thicknesses near or below the long period lead to the formation of standing cylinders or lamellae. A parallel arrangement of cylinders or lamellae can be found if the film thickness exceeds the long period. This effect could be seen in the BCP morphologies of dip coated films with thicknesses of 37 nm and 60 nm as shown in Figure 14a,b, respectively.

Shortened CNTs BM_4.5h was located parallel to the film surface and could not embed directly into the PB-rich phase in the BCP morphology when exhibiting predominantly standing cylinders or lamellae (Figure 14c). However, the visible CNTs, which had a length of ≈300 nm, were surrounded by the soft phase, i.e., there was an enrichment of the PB-rich phase around the CNTs. Such template effect has been discussed by Krishnamoorti and coworkers [43,44] for layered silicates governing the structure development of SEBS block copolymers. The BCP morphology of the thicker film exhibiting lying lamellae and individual CNTs with a length of about 100 nm were partly localized within the PB-rich phase (Figure 14d).

### 3.4. Stress-Strain Behavior

**Figure 15:**
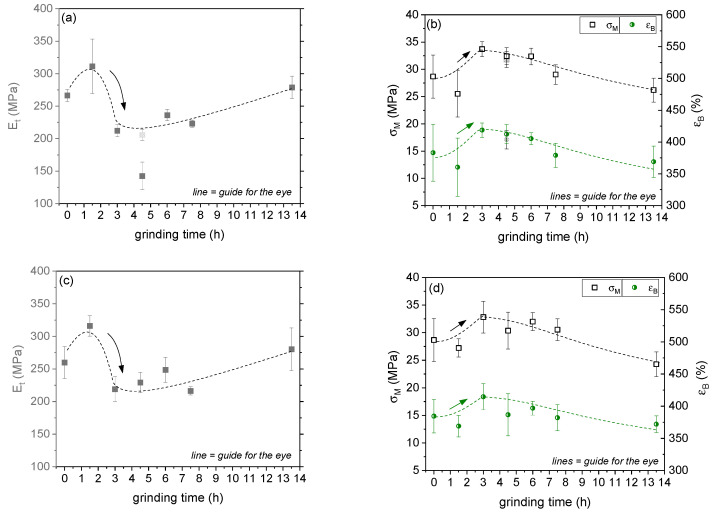
Young’s modulus (*E_t_*), tensile strength (*σ_M_*), and elongation at break (*ε_B_*) of solution cast BCP/CNT composites with 0.1 wt% CNTs (**a**,**b**) and 1 wt% CNTs (**c**,**d**) as function of grinding time.

The SB star block copolymer combines the properties of its hard and soft components, is transparent, and exhibits a ductile property profile with large elongation at break [39,45]. Averaged stress-strain diagrams of BCP/CNT composites containing various contents of neat NC7000 and ground CNTs BM_1.5h and BM_13.5h are presented in Figure S5, Supplementary Materials. The diagrams illustrate that the stress-strain behavior was largely independent of the CNT content and of the CNT treatment by ball milling. In Figure 15, the mechanical values Young’s modulus (*E_t_*), tensile strength (*σ_M_*), and elongation at break (*ε_B_*) for BCP/CNT composites with 0.1 wt% CNTs (Figure 15a,b) and for composites with 1 wt% CNTs (Figure 15c,d) are compared. Considering the standard deviation of the mechanical values, no significant changes can be discussed for the yield stress. However, grinding the CNTs for 1.5 h initially caused an increase in Young’s modulus from ≈260 MPa (for BCP/NC7000 composites and neat BCP) to ≈315 MPa, which could be observed both for composites with 0.1 wt% of CNTs (Figure 15a) and for composites with 1 wt% of CNTs (Figure 15c). This indicated a dependence on CNT properties and ball milling and thereof improved macrodispersion of CNTs for BM_1.5h (see Figure 9). For composites with CNTs ground for 3 h, a significant decrease in Young’s modulus of ≈30%, i.e., to 212 MPa and 219 MPa for composites with 0.1 wt% and 1 wt% CNTs, respectively, with a corresponding increase in elongation at break was exhibited. With increasing grinding time of CNTs up to 7.5 h, these mechanical values remained relatively constant. For composites with CNTs milled for 13.5 h, Young’s modulus, tensile strength, and elongation at break were again in the range of the mechanical values for the pure BCP.

**Figure 16:**
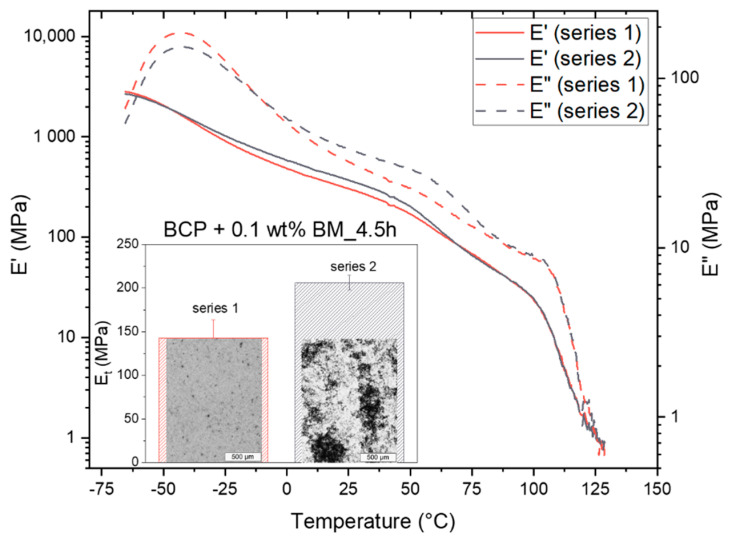
Specific characteristics of phase behavior (*E*′ and *E*″) and mechanical properties (*E_t_*) of BCP/CNT composites with 0.1 wt% BM_4.5h; insert diagram also presents transmission light microscopy images of the 0.3 mm thick solution cast films, visualizing CNT re-agglomeration in series 2 compared to well-dispersed CNTs in series 1.

However, a further distinct decrease in Young’s modulus could be observed for the BCP/CNT composite with 0.1 wt% BM_4.5h. Due to the strong shortening of these CNTs, they tended to localize in the PB-rich phase, which, as already discussed on the basis of the TEM and AFM images and DMA results, can lead to small changes in the (local) morphology and apparently causes softening of the material. Interestingly, a second series of the same BCP/CNT composition did not show such distinct decrease in Young’s modulus as marked by a grey symbol in Figure 15a. A more detailed comparison of the characteristics of these two series of BCP/CNT nanocomposites with 0.1 wt% BM_4.5h is illustrated in Figure 16. It could be observed that series 2 also did not exhibit such distinct decrease in E′ and E” as exhibited for series 1. The pictures, which are presented in the insert diagram of Young’s modulus (*E_t_*) in Figure 16, represent light microscopy images in transmission mode of solution cast films as prepared by an evaporation process (thickness ≈ 0.3 mm) for series 1 and series 2, respectively. These images visualize the differences in film quality between the two series. While series 1 showed a very good and homogeneous CNT dispersion resulting in a consistent slight transparency of the films, series 2 showed secondary agglomeration of CNTs during evaporation, resulting in a very inhomogeneous film quality with partly well-transparent regions and non-transparent areas with agglomerate formation. Such re-agglomeration process was also described in a previous publication [17]. Secondary agglomeration also occurred in films containing CNTs that were ground for shorter times than 4.5 h or not ground at all. Nanocomposites containing CNTs milled for longer than 4.5 h all exhibited homogeneous film quality and slight transparency of the films. These effects could only be characterized for nanocomposites with low CNT fractions of 0.1 wt%, and for composites with BM_7.5h and BM_13.5h, of 0.5 wt%, since at higher CNT fractions, the solution films no longer exhibited transparency and appeared completely black.

The significant differences in film quality, stiffness (*E_t_*), and phase behavior (*E*′ and *E*″) between two series of the same BCP/CNT composition can be explained by the specific properties of the CNTs BM_4.5h and the adjusted CNT concentration. It is likely that the grinding time of 4.5 h represents a critical grinding time at which optimal CNT shortening occurs and the fraction of highly compressed CNT agglomerates is still low, thus favoring good CNT dispersion as well as embedding of CNTs in the soft PB-rich phase (Figure 14c,d). Since the ball-milled CNT powder was not homogeneous but had a size distribution (see Figure 4), the very low CNT weights (2 mg for a fraction of 0.1 wt%) for series 1 and series 2 may have different characteristics in the size distribution, which then affected the film properties quite significantly. Moreover, the CNT concentration of 0.05 mg/mL is obviously close to the critical concentration above which the dispersion was dominated by agglomeration. This concentration is also called the dispersion limit which was already discussed for dispersions of MWCNTs NC7000 in polycarbonate–chloroform solutions [46] and has been determined for SWCNTs by other authors [47,48]. The very slight changes in concentration of the CNTs, which can occur due to minimal variations in the preparation, can thus already significantly change the dispersion behavior. In order to define the limits for stable and homogeneous CNT dispersion and selective CNT localization in BCP/CNT composites more precisely, it is reasonable to vary the grinding time in a smaller window of 3 h to 5 h in the future and to investigate the concentration ranges from 0.05 wt% to 0.5 wt% CNTs in more detail.

### 3.5. Electrical Properties

**Figure 17:**
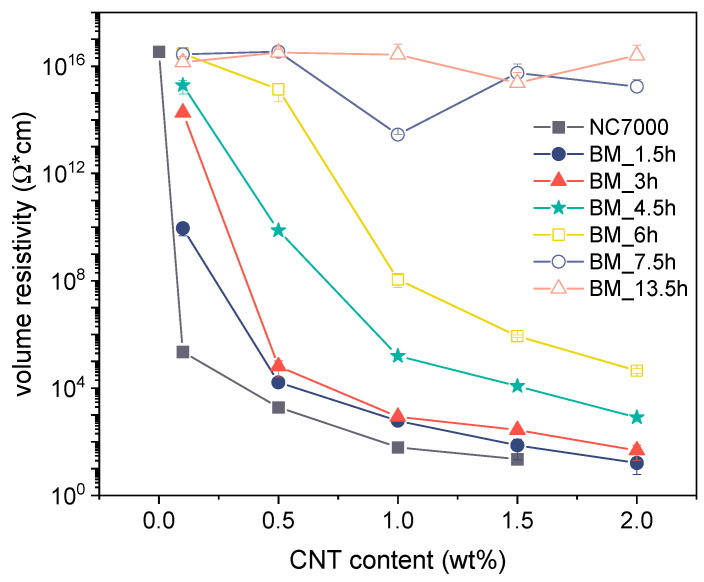
Volume resistivity of BCP/CNT composites as a function of CNT content.

Figure 17 presents the volume resistivity versus CNT content for BCP/CNT composites with different CNT size fractions. The electrical percolation threshold, which characterizes the formation of a continuous path of CNTs through the matrix and results in an abrupt change in electrical conductivity, was shifted to higher CNT content as the grinding time of CNTs increased. While composites containing neat NC7000 were already electrically conductive with CNT contents of 0.1 wt%, composites with CNTs ground for 7.5 h and 13.5 h were non-conductive within the investigated range up to 2 wt% CNTs. Due to the shortening of the CNTs by ball milling which resulted in a decreased aspect ratio, more particles were needed to form a percolated network structure. The conductivity of the CNT powders, which was twice as high for BM_4.5h and BM_6h as for the pure NC7000 (see Figure 6b), did not seem to have any influence on the conductivity of the composites, since the length of the CNTs and their dispersion primarily determine the percolation. As is generally known, a mixture of loose CNT agglomerates and dispersed CNTs is advantageous for achieving electrical percolation.

**Figure 18:**
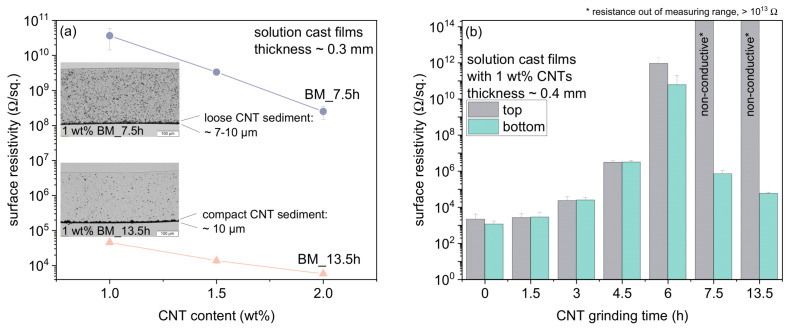
(**a**) Surface resistivity versus CNT content of the bottom of BCP/CNT composite films with a thickness of ≈0.3 mm containing CNTs ground for 7.5 h and 13.5 h, which formed a CNT sediment; (**b**) surface resistivity versus CNT grinding time from the top and from the bottom of BCP/CNT solution cast films with 1 wt% CNTs having a thickness of ≈0.4 mm.

As already discussed on the basis of the TLM images, a sedimentation process of CNT agglomerates took place in solution-mixed composites containing CNTs ground for 7.5 h and 13.5 h during the evaporation of the solvent. This led to the strong accumulation of CNTs at the bottom of the film. The top side was non-conductive, while the bottom side was electrically conductive or semi-conductive for composites with CNTs BM_13.5h or BM_7.5h, respectively (Figure 18a). As CNT agglomerates were more compressed at higher grinding times, these (BM_13.5h) sedimented more (faster) than less dense agglomerates (BM_7.5h) over the duration of the evaporation process. The sediment in composites with BM_13.5h was denser and more uniformly developed with a thickness of about 10 µm, while in composites with BM_7.5h, it was more loosely formed with a non-uniform thickness between about 7–10 µm (Figure 18a).

Figure 18b shows the surface resistivity at the top and bottom of BCP/CNT composite films containing 1 wt% CNTs as a function of grinding time. It should be noted that these films had a thickness of ≈0.4 mm, which is higher than those for the films otherwise used in the study. These dispersions were poured into smaller-diameter Petri dishes and thus the fill level was higher. The evaporation process therefore took more time. As a result, sedimentation of CNT agglomerates in composites with BM_7.5h and BM_13.5h could take place longer and the sediment could form more densely. This was mainly reflected in the values of surface resistivity for the composite with 1 wt% BM_7.5h, which was significantly lower (7.2 × 10^5^ Ω/sq.) at the bottom of the film with a thickness of ≈0.4 mm than for films with a thickness of ≈0.3 mm (3.7 × 10^10^ Ω/sq.). From the diagram in Figure 18b, it is also evident that CNT sedimentation already occurred in composites with BM_6h and caused small differences in surface resistivity for the top and bottom sides of the film.

Thus, by controlled introduction of shortened CNTs into BCP solutions and setting defined evaporation conditions, functional materials with special electrical properties could be produced with tailorable conductivities at the top and bottom of the film.

## 4. Conclusions

In this study, MWCNTs were ball-milled for 1.5 h to 13.5 h to reduce their length to the extent that localization occurs within the nanodomains of a nanostructured star-shaped SB block copolymer. An effective reduction in CNT length occurred between 3 and 6 h of grinding, which was also accompanied by an improvement in CNT dispersion in the block copolymer. The D50 value decreased more than eight-fold from 1341 nm for the untreated CNTs to a D50 value of 162 nm for the CNTs milled for 6h. Short CNTs were able to localize in the PB-rich phase as demonstrated by TEM and AFM studies. The electrical percolation threshold increased with increasing grinding time of CNTs, whereas for nanocomposites with CNTs ground for 7.5 h and 13.5 h, no significant reduction in volume resistivity with CNT addition occurred. In nanocomposites with CNTs milled for 7.5 h and 13.5 h, a sedimentation effect of compact CNT agglomerates occurred during the evaporation of the solution cast films. This resulted in the films having an insulating surface and an electrically conductive bottom side. The stress-strain behavior of the composites was hardly affected by the shortening of the CNTs. However, a distinct increase in Young’s modulus (*E_t_*) of ≈20% for composites containing CNTs ground for 1.5 h compared to composites with neat CNTs and to pure BCP was observed, which may be due to the improved macrodispersion of the ground CNTs. This was followed by a significant decrease in *E_t_* of ≈30% for nanocomposites with CNTs milled for 3 h to 7.5 h, with a slight increase in elongation at break. This was apparently related to the localization of short CNTs in the PB-rich phase, which was accompanied by changes in phase behavior and local morphology, leading to softening of the material. In summary, this study contributed to the elucidation of the properties of ball-milled CNTs and the dispersion and localization behavior of shortened CNTs in nanostructured BCPs. These findings on the interactions between BCP morphology and shortened CNTs provide a basis for further development of functional BCP nanocomposites, e.g., for use as sensing materials for the detection of gases or mechanical deformations, which is a part of current research.

## Data Availability

Data generated during the study were archived on the research institute’s internal server and can be provided by the corresponding author upon reasonable request.

## Supplementary Materials

The following supporting information can be downloaded at: https://www.mdpi.com/article/10.3390/polym14132715/s1, Supplementary Materials: Influence of CNT length on their dispersion, localization, and electrical percolation in a styrene–butadiene-based star block copolymer; Figure S1: SEM images of CNT powders: neat NC7000 (a) and CNTs ground for 1.5 h (b), 3 h (c), 4.5 h (d), 6 h (e), 7.5 h (f), and 13.5 h (g); Table S1: Polydispersity index (PDI) and particle size distribution of ground CNTs, measured by DLS; Figure S2: Transmission profiles of BCP/CNT dispersions for various CNT size fractions: neat NC 7000 (a) and CNTs ground for 1.5 h (b), 3 h (c), 4.5 h (d), 6 h (e), 7.5 h (f), and 13.5 h (g); measured using centrifugal separation analyzer for characterizing the sedimentation behavior of CNTs in BCP/toluene solution; Figure S3: Photographs of BCP/CNT dispersions after centrifugation containing 1 wt% of neat NC7000 (a) and CNTs ground for 1.5 h (b), 3 h (c), 4.5 h (d), 6 h (e), 7.5 h (f), and 13.5 h (g); Figure S4: AFM height and phase images of neat BCP (a, d), BCP with 1 wt% NC7000 (b,e), and BCP with 1 wt% BM_6h (c,f), respectively; Figure S5: Averaged stress-strain diagrams of BCP/CNT composites containing various filler content of NC7000 (a), BM_1.5h (b), and BM_13.5h (c).
