# Supplementary material for: Influence of CNT Length on Dispersion, Localization, and Electrical Percolation in a Styrene-Butadiene-Based Star Block Copolymer
Source: Polymers (Basel). 2022 Jul 2;14(13):2715. doi: 10.3390/polym14132715 (PMC9268902; doi:10.3390/polym14132715)
Supplement: Supplementary file 1 [file polymers-14-02715-s001.zip › polymers-1779497-supplementary.pdf]

polymers

MDPI

Supplementary Materials

Influence of CNT Length on Dispersion, Localization, and Electrical Percolation in a Styrene-Butadiene-Based Star Block Copolymer

Ulrike Staudinger [1,*], Andreas Janke [1], Christine Steinbach [1], Uta Reuter [1], Martin Ganß [2] and Oliver Voigt [1,†]

[1] Leibniz-Institut für Polymerforschung Dresden e.V., Hohe Str. 6, 01069 Dresden, Germany; andy@ipfdd.de (A.J.); steinbach@ipfdd.de (C.S.); reuter@ipfdd.de (U.R.); oliver.voigt@mvtat.tu-freiberg.de (O.V.)
[2] Material Research and Testing Institute (MFPA) at the Bauhaus-Universität Weimar, Coudraystraße 9, 99423 Weimar, Germany; martin.ganss@mfpa.de
* Correspondence: staudinger@ipfdd.de; Tel.: +49-351-4658-646
† Current Address: Institut Mechanische Verfahrenstechnik und Aufbereitungstechnik, Technische Universität Bergakademie Freiberg, Agricolastraße 1, 09599 Freiberg, Germany.

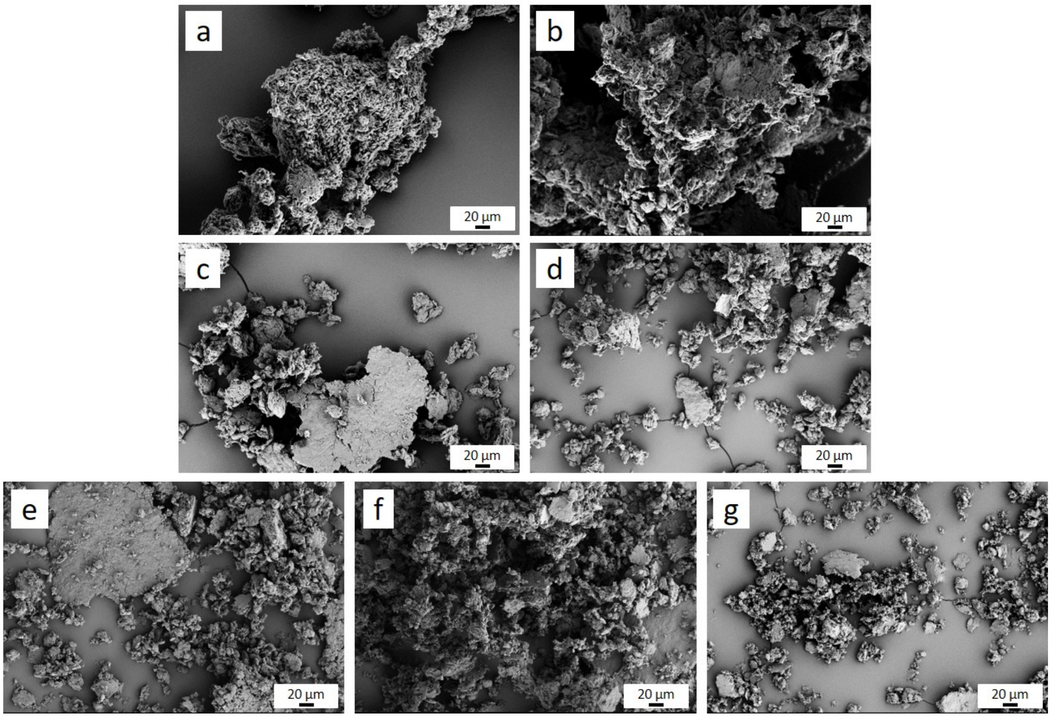

Figure S1. SEM images of CNT powders: neat NC7000 (a) and CNTs grinded for 1.5 h (b), 3 h (c), 4.5 h (d), 6 h (e), 7.5 h (f) and 13.5 h (g)

Table S1. Polydispersity index (PDI) and particle size distribution of grinded CNTs, measured by DLS.

**Sample**	**No.**	**PDI**	**Intensity***		**Volume***		**Number***	
			**size d (nm)**	**fraction (%)**	**size d (nm)**	**fraction (%)**	**size d (nm)**	**fraction (%)**
**CNT_BM_6h**	**a**	0.647	983.3	88.9	1021	97.0	939.8	9.3
			142.8	11.1	145.2	3.0	140.1	90.7
	**b**	0.738	1051	84.2	1134	94.7	951.8	7.9
			171.4	15.8	181.7	5.3	163.7	92.1
	**c**	0.594	1232	88.3	1307	97.2	1168	7.9
			163.6	11.7	167.8	2.8	161.4	92.1
	**d**	0.557	1239	86.3	1313	96.2	1174	7.3
			177.1	13.7	183.6	3.8	173.7	92.7
**CNT_BM_7.5h**	**a**	0.662	1102	90.0	1187	95.1	1008	16.8
			232.2	10.0	241.7	4.9	228.5	83.2
	**b**	0.696	1074	92.6	1144	96.8	998.5	20.6
			213.0	7.4	220.3	3.2	210.2	79.4
**CNT_BM_13.5h**	**a**	0.750	669.7	100.0	674.0	100.0	663.1	100.0
	**b**	0.605	702.6	100.0	709.7	100.0	678.5	100.0
	**c**	0.711	758.3	100.0	772.1	100.0	729.9	100.0

*Distribution analysis

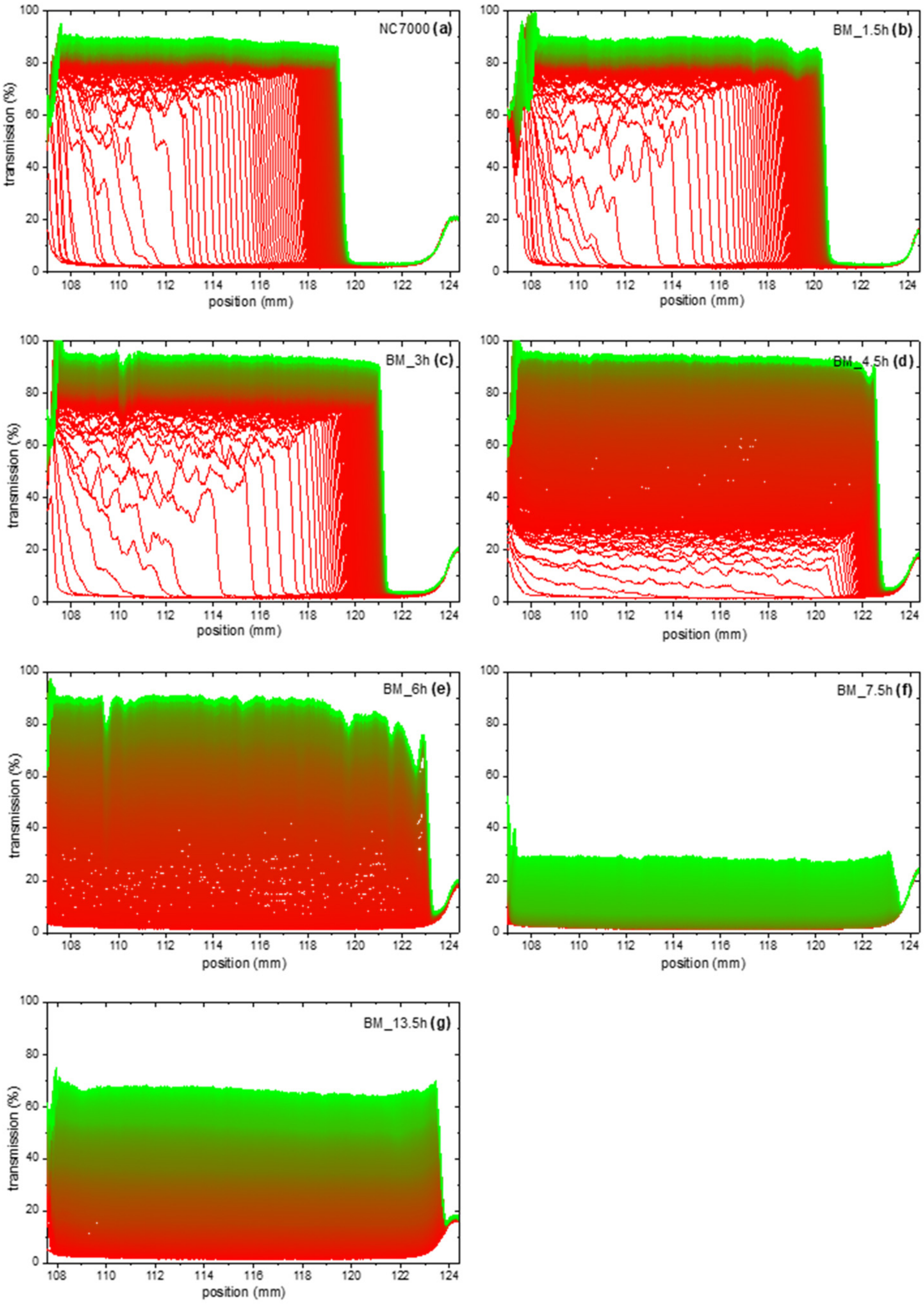

Figure S2. Transmission profiles of BCP/CNT dispersions for various CNT size fractions: neat NC 7000 (a) and CNTs grinded for 1.5 h (b), 3 h (c), 4.5 h (d), 6 h (e), 7.5 h (f) and 13.5 h (g); measured using Centrifugal Separation Analyzer for characterizing the sedimentation behavior of CNTs in BCP/toluene solution

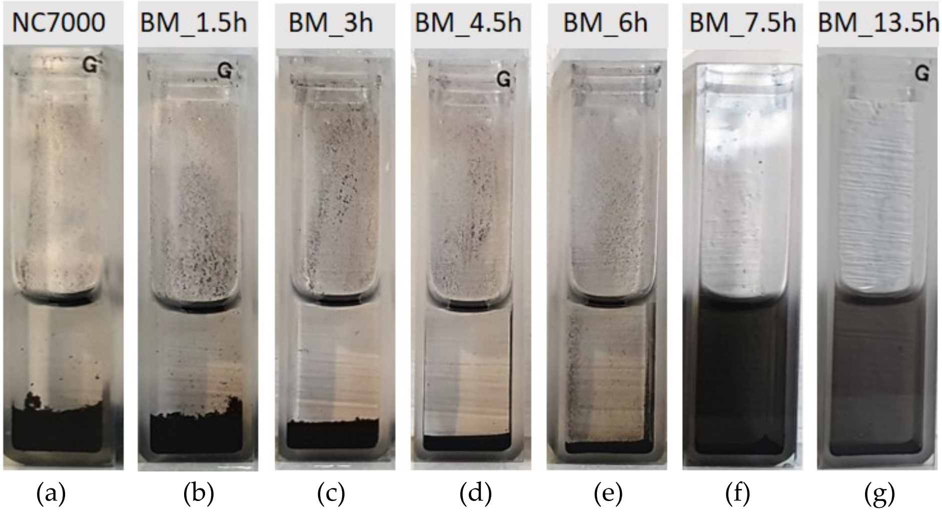

Figure S3. Photographs of BCP/CNT dispersions after centrifugation containing 1 wt% of neat NC7000 (a) and CNTs grinded for 1.5 h (b), 3 h (c), 4.5 h (d), 6 h (e), 7.5 h (f) and 13.5 h (g)

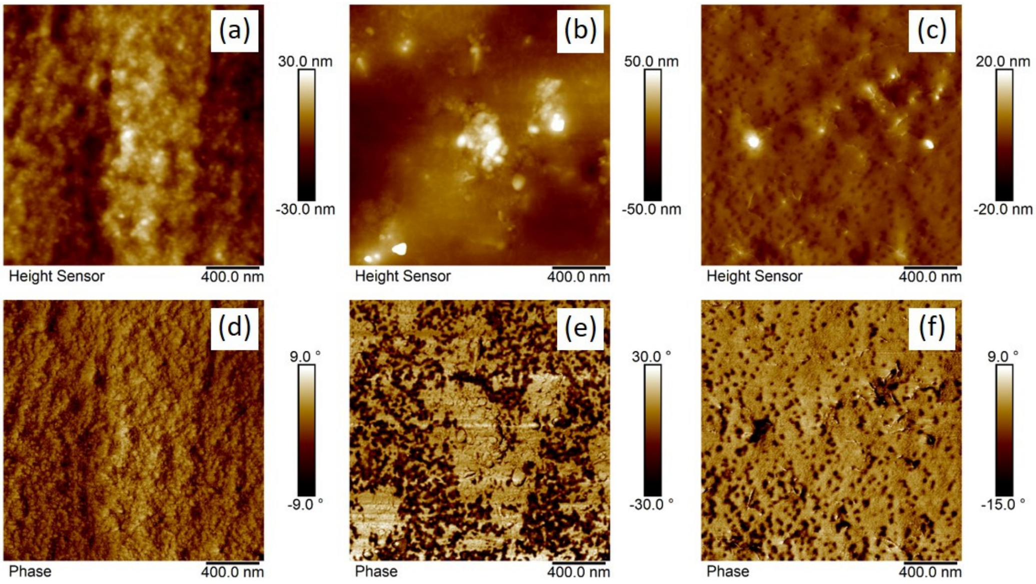

Figure S4. AFM height and phase images of neat BCP (a,d), BCP with 1 wt% NC7000 (b,e) and BCP with 1 wt% BM_6h (c,f), respectively

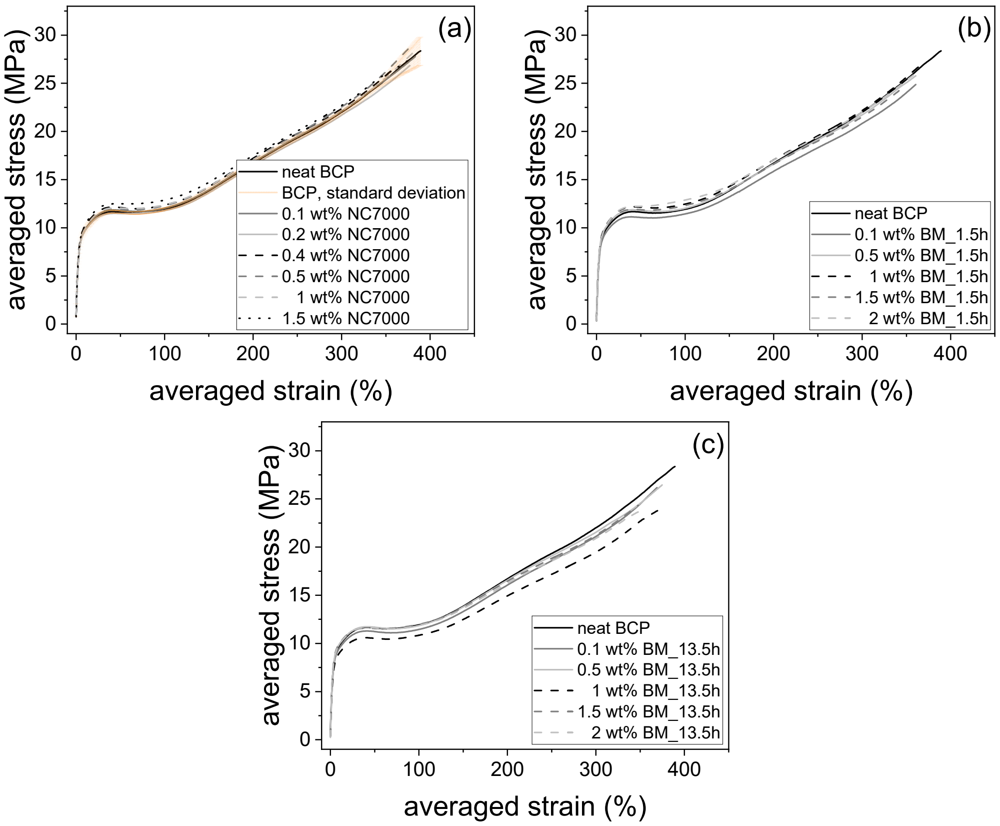

Figure S5. Averaged stress-strain diagrams of BCP/CNT composites containing various filler content of NC7000 (a), BM_1.5h (b) and BM_13.5h (c)
